# Glucocorticoid-Driven NLRP3 Inflammasome Activation in Hippocampal Microglia Mediates Chronic Stress-Induced Depressive-Like Behaviors

**DOI:** 10.3389/fnmol.2019.00210

**Published:** 2019-08-29

**Authors:** Xiujing Feng, Yuan Zhao, Tianyuan Yang, Manyu Song, Chaoran Wang, Yujie Yao, Honggang Fan

**Affiliations:** Heilongjiang Key Laboratory for Laboratory Animals and Comparative Medicine, College of Veterinary Medicine, Northeast Agricultural University, Harbin, China

**Keywords:** stress, glucocorticoid, NLRP3 inflammasome, hippocampal microglia, neuroinflammation, depressive-like behaviors

## Abstract

Chronic stress is a key risk factor for depression, and microglia have been implicated in the pathogenesis of the disease. Recent studies show that the Nod-like receptor protein 3 (NLRP3) inflammasome is expressed in microglia and may play a crucial role in depression. However, the mechanism of NLRP3 inflammasome activation in hippocampal microglia and its role in depressive-like behaviors remain poorly understood. In this study, rats were subjected to 6 h of restraint stress per day for 21 days to produce a model of stress-induced depression. Behavioral tests and serum corticosterone were used to assess the success of the model. Furthermore, HAPI cells were pretreated with dexamethasone (5 × 10^–7^ M) to assess stress-induced changes in microglial cells in culture. The microglial marker Iba-1, reactive oxygen species (ROS), nuclear factor kappa B (NF-κB) and key components of the NLRP3 inflammasome and its downstream inflammatory effectors (IL-1β and IL-18) were measured. Chronic stress induced depressive-like behavior, increased serum corticosterone levels and produced hippocampal structural changes. Chronic stress and dexamethasone both increased Iba-1 expression and ROS formation and also elevated levels of NF-κB, NLRP3, cleaved caspase-1, IL-1β and IL-18. After use of the NF-κB inhibitor BAY 117082 and knocked out NLRP3 *in vitro* decreased ROS formation and the expression of Iba-1, NF-κB and NLRP3 as well as levels of cleaved caspase-1, IL-1β and IL-18. These findings suggest that activation of the glucocorticoid receptor-NF-κB-NLRP3 pathway in hippocampal microglia mediates chronic stress-induced hippocampal neuroinflammation and depression-like behavior.

## Introduction

Depression is a common mental disease with high morbidity, recurrence and mortality and is a serious global health problem (Yang et al., 2019). Stress, especially chronic stress, is considered an important risk factor for depression, and it severely impairs cognition and learning and memory functions (Hayden et al., 2010). Chronic stress activates hypothalamic-pituitary-adrenal (HPA) axis, which results in persistent release of glucocorticoids throughout the brain (Rivier and Vale, 1983), especially the hippocampus (Zhao et al., 2015). The glucocorticoid receptor (GR), which is the main receptor for glucocorticoids, is highly expressed in the hippocampus (Joels, 2011). Furthermore, several studies have shown that the hippocampus, amygdala and the prefrontal cortex play major roles in depression (Price and Drevets, 2010). In particular, the hippocampus, a stress-sensitive limbic structure, is important for cognition and spatial memory, and these functions are impaired in depressive disorder (Austin et al., 2001). Thus, the hippocampus is intimately involved in the pathophysiology of depression.

Microglia are the resident immune cells in the CNS, and depression is increasing considered a microglial disease (Yirmiya et al., 2015). Furthermore, preclinical studies show that microglia activation is involved in the pathogenesis of depression (Bollinger et al., 2016). Accumulating evidence indicates that microglial cells are widely distributed in the hippocampus and prefrontal cortex, which are brain regions that have a critical role in the regulation of mood and behavior (Lawson et al., 1990; Drevets et al., 2008). Furthermore, hippocampal microglial activation promotes the release of inflammatory factors, which results in the disruption of neuroplasticity and cognitive impairment, thereby contributing to the development of depression (Walker et al., 2013; Singhal and Baune, 2017). Microglial activation is a key mediator of neuroinflammatory processes (Tronel et al., 2017), and neuroinflammation plays a crucial role in the pathogenesis of depression (Zhang C. et al., 2019). In particular, HPA axis hyperactivity induces the overproduction of brain pro-inflammatory cytokines through microglial activation (Brites and Fernandes, 2015), and this phenomenon is consistently observed in subjects with depressive disorders (Zou et al., 2018). However, the mechanisms underlying chronic stress-induced hippocampal microglia activation and neuroinflammation in depression remain unclear.

Recent studies show that the Nod-like receptor protein 3 (NLRP3) inflammasome and related pathways are associated with the pathogenesis of depression (Xu et al., 2016; Gao et al., 2018). NLRP3 inflammasome activation is observed in animal models of depression (Zhang Y. et al., 2015) as well as in depressive patients (Alcocer-Gomez et al., 2014). One study shows that the antidepressant mechanism of silymarin may be associated with inhibition of neuroinflammation and NLRP3 inflammasome activation in CUMS-induced depression, at least in part proves that NLRP3 inflammasome activation is reduced by antidepressant treatment, and accordingly, it is a potential new target for the development of antidepressant strategies (Ashraf et al., 2019). Studies have shown that activation of P2X7 receptor and the NLRP3 inflammasome in hippocampal glial cells mediates depressive-like behavior induced by chronic stress (Yue et al., 2017). However, whether the NLRP3 inflammasome is activated in hippocampal microglia during chronic restraint stress and depression remains unclear.

Reactive oxygen species (ROS) are important activators of inflammation mediated by the NLRP3 inflammasome (Zhou et al., 2011). Furthermore, nuclear factor kappa B (NF-κB)-induced oligomerization of NLRP3 with apoptosis-associated speck-like protein containing a CARD (ASC) and pro-caspase 1 forms the NLPR3 inflammasome (Gross et al., 2011). In response to stress, the activated NLRP3 inflammasome cleaves pro-caspase 1 to the mature caspase-1 p10 and p20. Subsequently, inactive pro-IL-1β and pro-IL-18 are converted into their active forms, IL-1β and IL-18 (Walsh et al., 2014). In addition, IL-1β, whose secretion is tightly controlled by the NLRP3 inflammasome, plays a critical role in the pathogenesis of depression (Park et al., 2015). Recent studies suggest that chronic glucocorticoid administration increases ROS levels in the brain (Uchihara et al., 2016) and promotes NF-κB transcription (Pace and Miller, 2009). Moreover, glucocorticoids upregulate both mRNA and protein levels of NLRP3 in macrophages and microglia (Frank et al., 2014). However, it remains unclear whether glucocorticoid-induced neuroinflammation and depressive behavior in the chronic restraint stress model of depression involves microglial NLPR3 inflammasome activation.

We hypothesized that chronic stress-induced neuroinflammation and depressive behaviors are associated with activation of the GR-NF-κB-NLRP3 signaling pathway in hippocampal microglia. In this study, we used the chronic restraint stress-induced model of depression as well as cultured HAPI cells treated with dexamethasone (DEX, a glucocorticoids hormone) to investigate whether chronic stress-induced hippocampal neuroinflammation is mediated by the GR-NF-κB-NLRP3 pathway, and which might be a new target and offer new perspectives on depression research. The results also provide a theoretical basis for the development of new antidepressants.

## Materials and Methods

### Animals

This study was approved by the ethical committee of Northeast Agricultural University (SRM-11), and experiments were carried out in accordance with the National Institutes of Health Guide for Care and Use of Laboratory Animals. For experiments, sixty adult (8 weeks old) male Wistar rats weighing 200–210 g (purchased from the Harbin Medical University Laboratory Animals Institutes, China) were housed at 22–24°C with appropriate humidity and a 12 h alternating light-dark cycle. Rats were housed in groups of 3 rats in standard polypropylene cages and *ad libitum* access to food and water. Animals were acclimated to the environment for 7 days before the beginning of the experiment.

### Experimental Design

**FIGURE 1:**
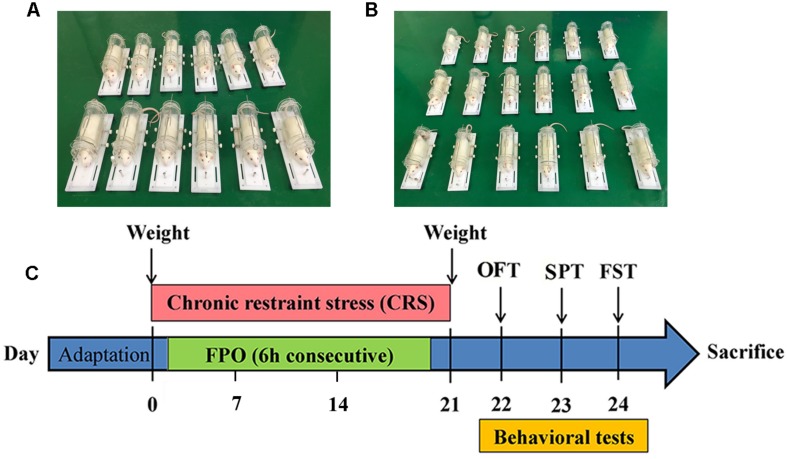
The Wistar rats were challenged with chronic restraint stress with 6 h each day during 21 consecutive days. **(A)** Images of restrained rats (*n* = 12) for behavioral testing, **(B)** Image of restrained rats (*n* = 18) for subsequent experiments, **(C)** Schematic diagram showing the schedule of Body weight, Fecal pellet output (FPO), Sucrose preference test (SPT), Open field test (OFT), Forced swimming test (FST).

Rats were randomly divided into two weight-matched groups: control group (C, *n* = 30) and chronic stress group (CS, *n* = 30). 12 rats in each group were used for behavioral experiments, and the remaining 18 were used for subsequent experiments. The chronic restraint stress procedure was performed between 9:00 and 15:00, as previously described (Zhao et al., 2013; Seo et al., 2017). Briefly, except rats in C group, all rats were daily restrained into a transparent plexiglass tube (26 cm long and 8 cm in diameter) for 6 h over 21 consecutive days (Figures 1A,B). During CS group rats were restrained, the rats in the C group stay in their home cages without water and food. After stress, rats in the C and CS groups were given conventional feed and free drinking water. The body weights in the C and CS group were measured on the 0, 7, 14, and 21 days. The behavioral experiments started on day 22 to 24 after 21 consecutive days restrained. At least 24 h between each behavior (Figure 1C).

### Fecal Pellet Output

After 6 h of restraint stress, the number of pellets released during restraint in the CS rats was collected. Considering that rat isolation may cause a stress response, 3 rats in the C group were kept in a cage, and the fecal output of the C group represents the mean value for a group.

### Behavioral Tests

#### Open Field Test

The open field test (OFT) was performed as previously described (Lee et al., 2014). The open-field box (100 cm × 100 cm × 40 cm) is made of wood and painted black inside. The bottom of the box is evenly divided into 25 squares. A camera was placed above the box to track and record rat performance. On day 22, rats were placed in the center of the open field box, and the rats’ behavior was monitored for 3 min, including total distance, average speed, number of crossings and number of rearing. The device was cleaned with 70% ethanol thoroughly after each trial. Open field test was analyzed and recorded by Super Maze software (Shanghai Softmaze Information Technology Co., Ltd., Shanghai, China).

#### Sucrose Preference Test

The sucrose preference test (SPT) was improved on the basis of predecessors (Overstreet, 2012). In a word, after deprivation of food and water for 12 h, rats were domesticated for 12 h, as two 1% sucrose bottles for 6 h, and then one of the 1% sucrose bottles was replaced with tap water for the next 6 h. On the day of the experiment, rats were given a bottle of 200 ml tap water and a bottle of 200 ml 1% sucrose solution for 12 h (6 h light/6 h dark). After 6 h of testing, change the position of the two bottles to avoid position preference. Percentage (sucrose intake/sucrose intake plus water intake) is expressed as sucrose preference.

#### Forced Swimming Test

The forced swimming test (FST) is performed as previously described and used to assess depressive-like behavior in animal models (Bogdanova et al., 2013; Lee et al., 2014). In this study, rats were placed individually into a vertical plexiglass cylinder measuring 45 cm height and 20 cm diameter containing water at 25 ± 1°C, and the cylinder water depth of 30 cm. The swimming sessions consisted 20 min and the lasted 5 min was recorded as test session. The FST sessions in 5 min were recorded by a video camera for later analysis. After completion of the FST, the rats were removed from the water, well dried with paper towels, and returned to their warm cages.

### Blood and Tissue Samples Collection

On day 21, at the end of the last chronic restraint stress, except for the rats that performed the behavioral experiments, the remaining rats were immediately anesthetized with isoflurane (Yipin Pharmaceutical Co., Ltd., Hebei, China). Then blood samples were collected quickly by heart puncture, and the supernatant was collected by centrifugation at 3000 rpm for 10 min at 4°C and stored at −80°C use for corticosterone assay. Simultaneously, brains were removed, brains (*n* = 6) per group were placed in 10% paraformaldehyde for hematoxylin and eosin staining (H&E) and immunohistochemistry (IHC). Hippocampal areas were isolated, hippocampus (*n* = 6) per group were placed in 3% glutaraldehyde to observe the changes of the ultrastructure of the hippocampus. Hippocampus (*n* = 6) per group were removed and immediately measured the concentration of ROS in the hippocampus, the rest of hippocampus were stored at −80°C for subsequent experiments.

### Histological and Ultrastructural Observations

Once the hippocampus was fixed, hippocampus sections (3 μm) were fixed in formalin for at least 24 h and embedded in paraffin prior to examination. The hippocampus sections were then stained using H&E (WUHAN XINXINJIALI Bio-tech Co., Ltd., Wuhan, China). After these procedures, sections were observed with light microscopy (TE2000, Nikon, Japan) and the camera (Canon, Tokyo, Japan) with the software was used for image capturing.

The hippocampus was cut into 1 mm^3^ blocks, fixed in 3% glutaraldehyde for 48 h. The blocks were post fixed with 1% osmium tetroxide for 2 h. Afterward, the blocks were dehydrated in a graded series of acetone (50, 70, 90, and 100%) for 10 min each times. Then embedded in fresh pure Epon812 resin and allowed to polymerize at 60°C for 2 h. Samples were sectioned (60 nm) stained with lead citrate during 5 min. Samples were viewed and photographed with a transmission electron microscope (TEM, Tecnai-G212, FEI Company, Netherlands).

### Serum Corticosterone Assay

According to the kit instructions, the serum corticosterone levels were measured using Corticosterone ELISA Kit (Nanjing Jiancheng Bioengineering Institute, Nanjing, China).

### Immunohistochemistry Staining

After hippocampus tissue embedded in paraffin, 3 μm thick sections were prepared for IHC. Dewaxing with xylene, hydration with different degrees of ethanol and incubation with 3% H_2_O_2_ blocked the production of endogenous peroxidase. After incubation with goat serum, primary antibody anti-GR (1:400, Bioss antibodies, Beijing, China) was applied in blocking solution overnight at 4°C. After incubated with HRP- conjugated goat anti-rabbit IgG (1:100, Beyotime Biotechnology, Shanghai, China) for 30 min, and then reacted with DAB substrate for 5 min. The sections were counterstained with hematoxylin for 3 min. Images were captured using an Olympus microscope.

### Cell Culture and Drug Treatments

HAPI cells were purchased from the Cell Bank of the Chinese Academy of Sciences (Shanghai, China). Cells were cultured in DMEM medium (HyClone) supplemented with 10% FBS (BI), penicillin/streptomycin (100 U/mL; 100 μg/mL) at 37°C in 5% CO_2_ atmosphere. The HAPI cells were seeded in 6-well plates or 96-well plates with 8 × 10^3^ cells/well and the culture medium was changed daily. The cell experiments were grouped as follows:

**Control group (CON):** The cells were incubated in complete medium without any treatment.
**Dexamethasone group (DEX):** The cells were incubated in complete medium for 24 h with 5 × 10^–7^ M dexamethasone (Sigma-Aldrich, San Francisco, CA, United States, D4902, ≥97%, dissolve in ethanol).
**NF-κB P65 inhibitor group (BAY):** The cells were pretreated with 10 μM BAY117082 (Selleck.cn, Shanghai, China) for 30 min before incubated in complete medium for 24 h with 5 × 10^–7^ M dexamethasone.
**NLRP3 knock out group (sgNLRP3):** The NLRP3 knock-out cells were incubated in complete medium for 24 h with 5 × 10^–7^ M dexamethasone.
**Vehicle group (E):** The cells were incubated in complete medium for 24 h with ethanol. The final ethanol concentration did not exceed 0.2%.

### CRISPR/Cas9 Mediated Genome Editing

NLRP3 knock-out cells were generated using CRISPR/Cas9 technology. HAPI cells were seeded (5 × 10^3^ cells/well) in 6-well plates and grown in DMEM supplemented with 10% FBS for 24 h at 37°C with 5% CO_2_. The Cas9 and gRNA expressing plasmid PX459 V2.0 (gift from Linlin Li, Lanzhou Veterinary Research Institute, China) was used for gene editing. The CRISPR target sites were: ACGCTAATGATCGACTTCAA (NLRP3). HAPI were transfected with 500 ng plasmid and 1.5 μL of Lipofectamine 2000 in Opti-MEM (Thermo Fisher Scientific) according to manufacturer’s instructions. The transfection was stopped after 6 h by replacing the medium with fresh complete serum containing medium. The cells were selected 5 days after transfection with puromycin (1 mg/mL, LEAGENE, Beijing, China) and doxycycline (10 μg, LEAGENE, Beijing, China). Monoclonal build stable cell lines. The phenotype was confirmed by Western blot.

### Cell Counting Kit-8 (CCK-8) Assay

Cell viability was measured by using the CCK8 assay (Beyotime Institute of Biotechnology, Suzhou, China). To assess the effects of dexamethasone on cell proliferation, the cells were incubated in DMEM medium for 24 h at the concentration gradient from 5 × 10^–6^ M to 1 × 10^–8^ M. 10 μL of CCK-8 solution reagent was added to 100 μL of culture medium in each well. The absorbance of each well was read at a wavelength of 450 nm on a BioTek microplate reader (BioTek Instruments, Thermo Fisher Scientific, Winooski, VT, United States).

### LDH Release Assay

Released LDH in culture supernatants from damaged cells was measured with LDH assay kit (Beyotime Biotechnology, Nantong, China) by following manufacturer’s instruction. Determination of absorption of samples at 490 nm using a BioTek microplate reader.

The calculation of % cytotoxicity followed the below equation, these values were subsequently normalized and expressed as a percentage of control.

% Cytotoxicity: [Experimental (OD490) − Blank (OD490)] × 100/[Maximum LDH release (OD490) − Blank (OD490)]

### Measurement of Intracellular ROS Accumulation

Intracellular ROS accumulation was determined by an ROS assay kit (Beyotime Biotechnology, Shanghai, China) that utilizes DCFH-DA as a fluorescent probe. After dexamethasone treatment, cells were incubated with 10 mM of DCFH-DA at 37°C for 30 min. Intracellular ROS were determined by a fluorescence microscope at an excitation wavelength of 488nm and an emission wavelength of 525 nm.

### Immunofluorescence Staining

HAPI cells samples were prepared for immunofluorescence (IF) assays. The primary antibodies were anti-p-NF-κB P65 (1:100, Cell Signaling Technology, Danvers, MA) and anti-Iba-1 (1:100, Abcam, Cambridge, United Kingdom). Then the slides were mounted by using DAPI (Goodbio Technology, Co., Ltd., Wuhan, China). Images were acquired with a Nikon Eclipse Ni inverted microscope (TE2000, Nikon, Japan).

### Western Blot Analysis

Equal amounts of protein samples (28 μg) were subjected to a SDS-PAGE and transferred to PVDF membranes. Membranes were blocked in 5% milk in TBST for 2 h followed by incubation with the following primary antibodies as follows: anti-p-NF-κB P65 (1:1000, Cell Signaling Technology), anti-NF-κB P65 (1:1000, Cell Signaling Technology), anti-Iba-1 (1:1000, Abcam), anti-β-actin (1:7500, Bioss antibodies), anti-GR (1:1000, Bioss antibodies), anti-NLRP3 (1:1500), anti-ASC (1:500), anti-pro-caspase 1 (1:500), anti-caspase-1 p20 (1:750), anti-pro-IL-1β (1:10000), anti-IL-1β (1:750), anti-IL-18 (1:1000) and anti-Lamin B (1:750) both from Wanlei Biotechnology (Shenyang, China). After three times washing with TBST, the membranes were incubated with appropriate secondary antibody. The protein bands were visualized by the ECL detection system (Thermo Scientific, Waltham, MA, United States) and quantified using Image J software. Nuclear and Cytoplasmic Protein Extraction Kit (Applygen Technologies, Co., Ltd., Beijing, China) was used to extract the cytoplasmic/nuclear proteins of NF-κB P65 according to the manufacturer’s protocol.

### Statistical Analysis

Data were expressed as mean ± SEM (standard error means). Unpaired two-tailed Student’s *t*-test was used to two groups’ comparison. One-way analysis of variance (ANOVA) followed by Tukey’s *post hoc* test was used to multi-group comparison. Statistical analysis and Graphs were made using PASW Statistics 18 software (SPASS, IL, United States) and GraphPad Prism 8 software (GraphPad Software Inc., San Diego, CA, United States), respectively. IHC intensity was quantified with Image-Pro Plus software (Media Cybernetics, Rockville, MD, United States). *P* < 0.05 was considered to be statistically significant.

## Results

### Chronic Stress Affects Body Weight and Fecal Output and Causes Behavioral Deficits in Rats

**FIGURE 2:**
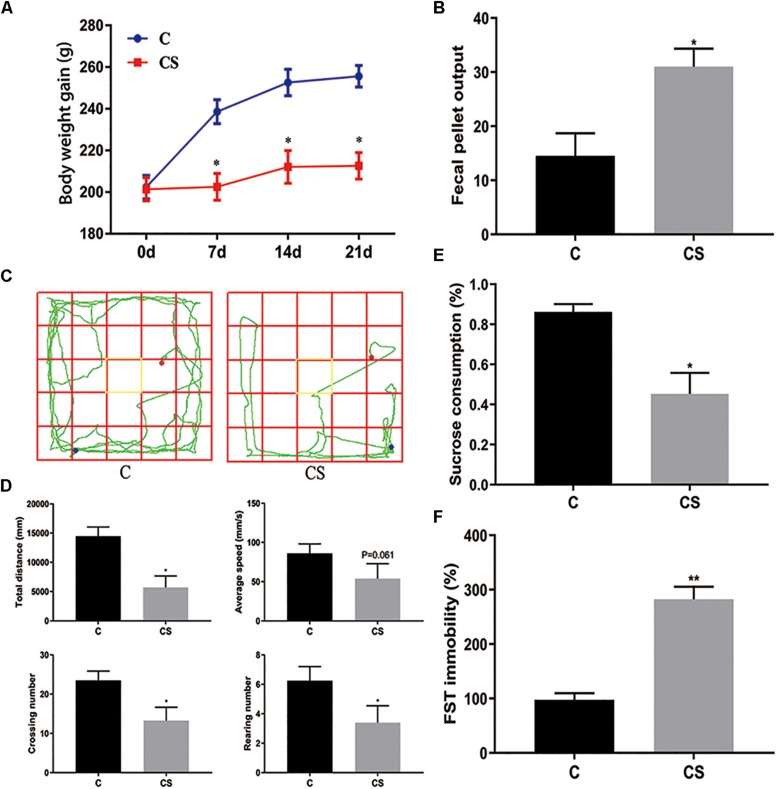
Chronic stress induced depression-like behavior in rats. **(A)** Body weight gain, **(B)** Fecal Pellet Output, **(C)** OFT track diagram, **(D)** OFT data analysis, **(E)** Sucrose Preference Test (SPT), **(F)** Forced swimming test (FST). Values are presented as mean ± SEM (*n* = 12). ^∗^*P* < 0.05, ^∗∗^*P* < 0.01 versus C group. Student’s two-tailed *t*-test **(A,B,D–F)**.

The body weight of rats was measured every 7 days (Figure 2A). Compared with the C group, weight gain was significantly reduced in the CS group. In addition, we assessed the physiological response to restraint stress by measuring daily fecal output. As shown in Figure 2B, the number of feces pellets was increased in the CS group. To verify the success of the model, we conducted behavioral experiments. In the OFT (Figures 2C,D), the total distance traveled, the number of crossings and rearing times in the CS group were significantly reduced, with no difference in average speed. In the SPT (Figure 2E), sucrose consumption in the CS group was reduced. In the FST (Figure 2F), immobility time in the CS group was increased significantly, indicating behavioral despair. These results suggest successful production of the rat model of depression.

### Chronic Stress Results in Histopathological and Ultrastructural Changes in the Hippocampus

**FIGURE 3:**
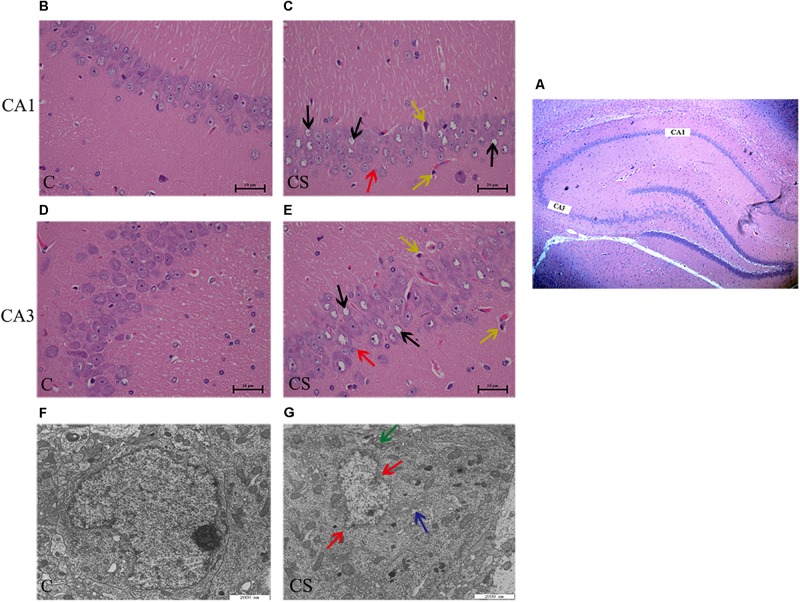
Chronic stress caused hippocampus microstructure and ultrastructure injury. **(A)** CA1 and CA3 regions of hippocampal (× 40), **(B,D)** C group showed the normal cells morphology in CA1 and CA3 region, **(C,E)** CS group showed the structures of neuronal cells were mussy and the cells morphology changed in CA1 and CA3 region. The red arrows (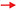) represent neurons displayed an irregular arrangement, enlarged pericellular spaces, the black arrows (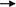) represent vacuolization of nerve cells and disappearance of nuclei, the yellow arrows (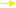) represents neuronal pyknosis and the coloring became deeper (×400, *n* = 6). **(F)** Ultrastructure of the hippocampus in C group, **(G)** Pathologic presentation of the ultrastructure of the hippocampus in CS group. (Scale bar = 2 μm, *n* = 6). The red arrows (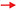) represent nucleus contraction, local rupture, the green arrows (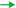) represent mitochondrial swelling and the blue arrows (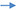) represent mild dilatation of endoplasmic reticulum.

In the CA1 and CA3 regions of the hippocampus (Figure 3A), the neurons were arranged in an orderly manner, with clear profiles and distinct nuclei and nucleoli in the C group (Figures 3B,D). Hippocampal neurons displayed an irregular arrangement, enlarged pericellular spaces (red arrows), an unclear nuclear structure, the absence of clearly visible nucleoli (black arrows), and neuronal pyknosis (yellow arrows) in the CS group (Figures 3C,E).

Transmission electron microscopy showed that, in the C group (Figure 3F), the nuclear membrane was complete, without evidence of abnormalities in the mitochondria, endoplasmic reticulum or other organelles. In the CS group (Figure 3G), the hippocampus showed signs of pathological changes, including nuclear shrinkage, rupture of the nuclear membrane (red arrows), swelling/degeneration of mitochondria (green arrows) and disappearance of the crests and mild dilation of the endoplasmic reticulum lumen (blue arrows).

### Chronic Stress Induces Glucocorticoid Release and ROS Production

**FIGURE 4:**
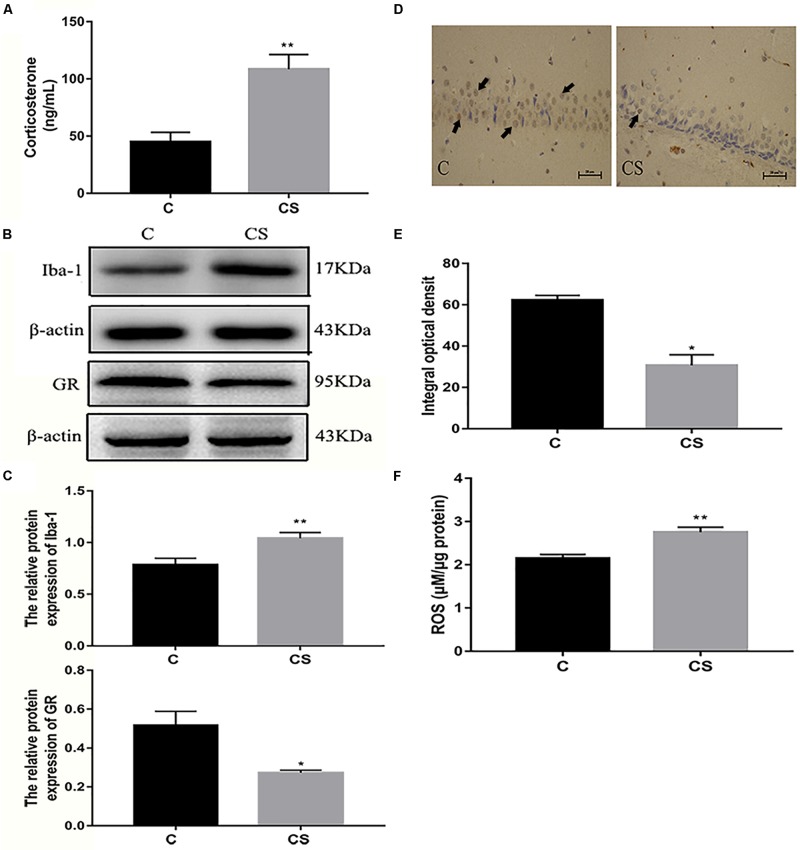
Chronic stress induced the increase of glucocorticoid level, ROS content and Iba-1 expression in hippocampus of rats. **(A)** Serum corticosterone levels, **(B**) The GR and Iba-1 expressions in hippocampus, **(C)** Quantitative analysis of GR and Iba-1 in hippocampus, **(D)** Immunohistochemistry (IHC) of GR in hippocampal CA1 cells, the black arrows (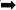) represents positive cells, **(E)** The percentages of GR positive in hippocampal CA1 cells, **(F)** The content of ROS in hippocampal. Data are expressed as mean ± SEM (*n* = 6). ^∗^*P* < 0.05, ^∗∗^*P* < 0.01 versus C group. Scale bar = 20 μm. Student’s two-tailed *t*-test **(A,C,E,F)**.

We speculated that chronic restraint stress would induce corticosterone release, which can be used as an index of HPA activation. Compared with the C group, serum corticosterone levels were increased in the CS group (Figure 4A). Activation of the HPA axis indicates that the rats did not adapt to daily exposure to the homotypic stressor. The expression of Iba-1 in the hippocampus was significantly higher in the CS group compared with the C group, while the expression of GR in the hippocampus was significantly reduced in the CS group compared with the C group (Figures 4B,C). Immunohistochemical analysis showed that the percentage of GR-positive cells (black arrows) was reduced in the hippocampal CA1 region in the CS group compared with the C group (Figures 4D,E). Furthermore, ROS content was significantly increased in the hippocampus in the CS group compared with the C group (Figure 4F).

### Chronic Stress Induces NF-κB and NLRP3 Inflammasome Activation in the Hippocampus

**FIGURE 5:**
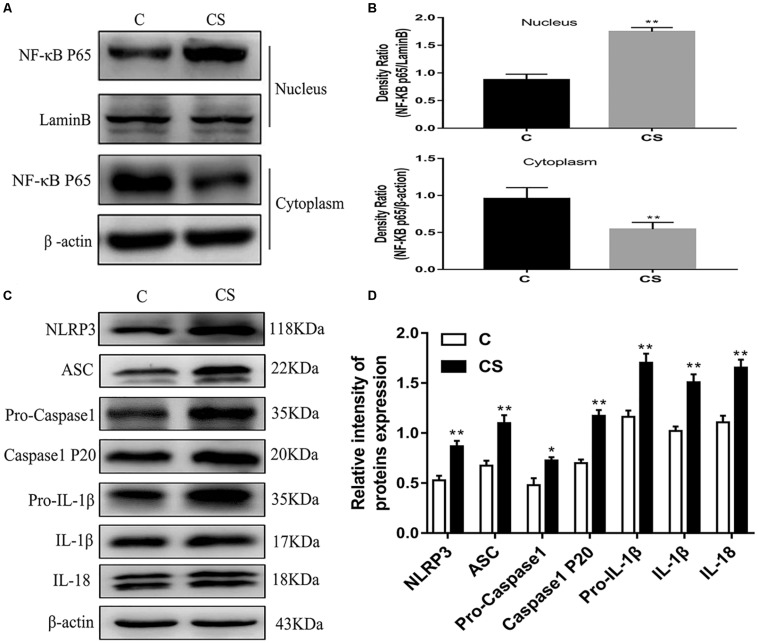
Chronic stress induces NF-κB p65 and NLRP3 inflammasome pathway in the hippocampus. **(A)** Cytoplasmic and nuclear NF-κB p65 expression in hippocampus, **(B)** Quantitative analysis of cytoplasmic and nuclear NF-κB p65 in hippocampus, **(C)** NLRP3 inflammasome components expression in the hippocampus, **(D)** Quantitative analysis of and NLRP3 inflammasome components in the hippocampus. Data are expressed as mean ± SEM (*n* = 6). ^∗^*P* < 0.05, ^∗∗^*P* < 0.01 versus C group. Student’s two-tailed *t*-test **(A,C)**.

To investigate whether NF-κB and the NLRP3 inflammasome are activated in the hippocampus of rats exposed to chronic restraint stress, we measured the protein expression levels of NF-κB and NLRP3 inflammasome components. Nuclear NF-κB p65 was significantly increased and cytoplasmic NF-κB p65 was significantly decreased in the CS group compared with the C group (Figures 5A,B). The expression levels of NLRP3 inflammasome components (NLRP3, ASC and pro-caspase 1) as well as caspase-1 p20, IL-1β and IL-18 were significantly increased in the CS group compared to the C group, (Figures 5C,D). These results suggest that chronic stress induces NF-κB p65 expression and nuclear translocation, activates the NLRP3 inflammasome and stimulates the production of IL-1β and IL-18.

### HAPI Cell Morphology and Optimum Concentration of Dexamethasone

**FIGURE 6:**
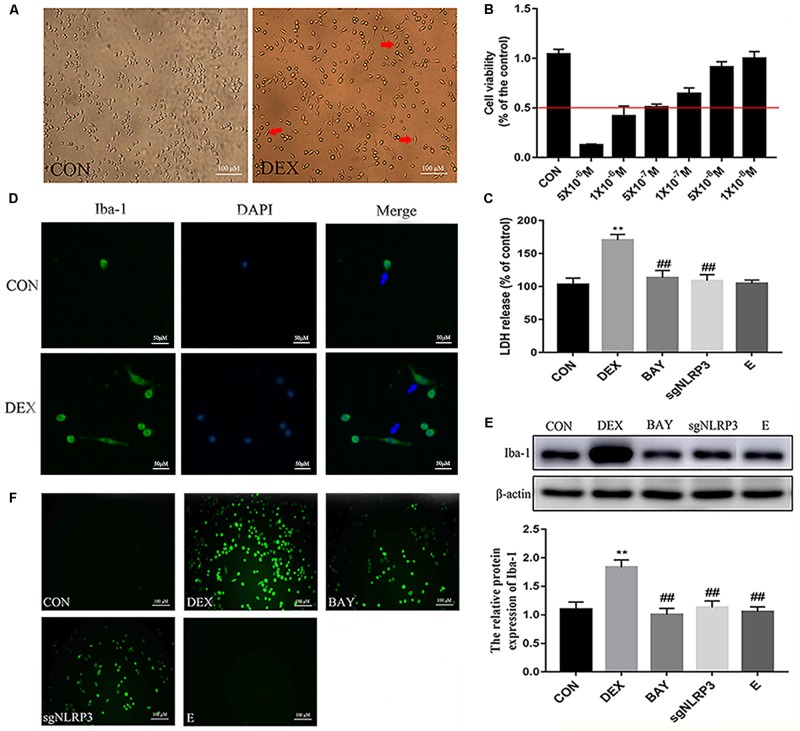
Dexamethasone increased LDH and ROS levels and activation HAPI cells. **(A)** HAPI cells morphology (× 200), the red arrows (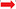) represents cells become ramified and some are star shaped with short thick processes, **(B)** CCK8 was used to detect cell viability after treatment with dexamethasone at different concentrations in HAPI cells, **(C)** LDH release in HAPI cells of different groups [F(4,15) = 40.81 *P* < 0.01], **(D)** Microglia marker Iba-1 was measured by Immunofluorescence (× 400), the blue arrows (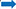) represents activated microglia, **(E)** The protein expression and quantitative analysis of Iba-1 [F(4,15) = 37.52 *P* < 0.01], **(F)** ROS levels in HAPI cells of different groups (× 200). Data are expressed as mean ± SEM. ^∗∗^*P* < 0.01 versus CON group; ^##^*P* < 0.01 versus DEX group. One-way ANOVA followed by Tukey’s *post hoc* test **(C,E)**.

HAPI cell morphology is shown in Figure 6A. HAPI cells in the CON group were mostly round, with high refraction and small dark nuclei. In contrast, cells in the DEX group were ramified, some with a star shaped and short thick processes (red arrows). To evaluate the optimum concentration of dexamethasone for HAPI cells, the CCK8 assay was performed. HAPI cells were exposed to dexamethasone at different concentrations (5 × 10^–6^ M, 1 × 10^–6^ M, 5 × 10^–7^ M, 1 × 10^–7^ M, 5 × 10^–8^ M, 1 × 10^–8^ M) for 24 h. As shown in Figure 6B, the 5 × 10^–7^ M concentration resulted in ∼50% viability.

### Dexamethasone Increases LDH and ROS Levels in HAPI Cells

To further explore changes in cell viability, we examined LDH released into the cell culture medium. As shown in Figure 6C, LDH release was significantly increased in the DEX group, while it was significantly decreased in the BAY and sgNLRP3 groups. The expression of the microglia marker Iba-1 was investigated by immunofluorescence (Figure 6D) and western blot (Figure 6E). After dexamethasone exposure, Iba-1 expression was increased in HAPI cells (blue arrows), while it was significantly decreased in the BAY and sgNLRP3 groups. We also evaluated the levels of ROS in HAPI cells by immunofluorescence (Figure 6F). ROS levels were significantly increased in the DEX group, while they were significantly reduced in the BAY and sgNLRP3 groups.

### Dexamethasone Activates NF-κB/NLRP3 Inflammasome Pathway in HAPI Cells

**FIGURE 7:**
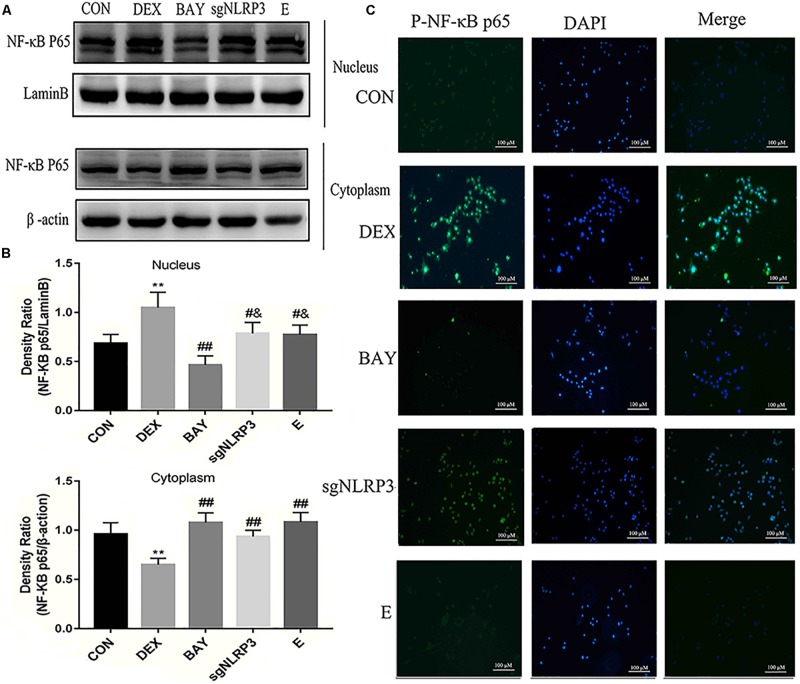
Dexamethasone exposure increased the expression of NF-κB p65 and promoted it translocate into nucleus in HAPI cells. **(A)** Cytoplasmic and nuclear NF-κB p65 expression in HAPI cells, **(B)** Quantitative analysis of cytoplasmic and nuclear NF-κB p65 in HAPI cells [nucleus, F(4,15) = 13.79 *P* < 0.01; cytosolic, F(4,15) = 15.01 *P* < 0.01], **(C)** Immunofluorescence detected nuclear localization of NF-κB p65 in HAPI cells. Green indicates NF-κB p65 and blue indicates nuclear. Data are expressed as mean ± SEM (×200). ^∗∗^*P* < 0.01 versus CON group; ^#^*P* < 0.05, ^##^*P* < 0.01 versus DEX group; ^&^*P* < 0.05 versus BAY group. One-way ANOVA followed by Tukey’s *post hoc* test **(B)**.

NF-κB may function as the upstream transcriptional activator of NLRP3. To further clarify the role of the NF-κB-NLRP3 inflammasome pathway in microglial, we used the NF-κB p65 inhibitor BAY117082 and knocked out the NLRP3 gene using the CRISPR/Cas9 technique. Compared with the CON group, nuclear NF-κB p65 levels were increased, while cytoplasmic NF-κB p65 expression decreased in the DEX group. Compared with the DEX group, nuclear NF-κB p65 was decreased, while cytoplasmic NF-κB p65 was increased in the BAY, sgNLRP3 and E groups. Compared with the BAY group, nuclear NF-κB p65 was increased, while cytoplasmic NF-κB p65 was unaffected in the sgNLRP3 and E groups (Figures 7A,B). Immunofluorescence analysis showed that after dexamethasone treatment, NF-κB p65 translocated to the nuclei in HAPI cells (Figure 7C). In the BAY group, nuclear entry of NF-κB p65 was reduced compared with the CON group. In the sgNLRP3 group, NF-κB p65 translocation into the nucleus was significantly decreased compared with the DEX group, while there was no significant difference from the CON group.

**FIGURE 8:**
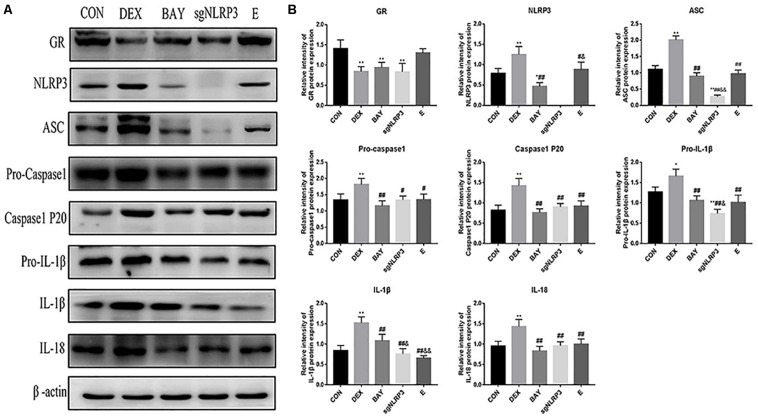
Dexamethasone exposure increased the expression of NLRP3 inflammasome pathway in HAPI cells. **(A)** The protein expressions of GR and NLRP3 inflammasome components and its downstream inflammatory factors, **(B)** Quantitative analysis of GR [F(4,15) = 11.13 *P* < 0.01] and NLRP3 inflammasome components NLRP3 [F(4,15) = 47.79 *P* < 0.01], ASC [F(4,15) = 135.38 *P* < 0.01], pro-caspase 1[F(4,15) = 8.83 *P* < 0.01], caspase-1 p20 [F(4,15) = 17.27 *P* < 0.01] and its downstream inflammatory factors pro-IL-1β [F(4,15) = 22.85 *P* < 0.05], IL-1β [F(4,15) = 29.09 *P* < 0.01], IL-18 [F(4,15) = 12.76 *P* < 0.01]. Data are expressed as mean ± SEM.^∗^*P* < 0.05, ^∗∗^*P* < 0.01 versus CON group; ^#^*P* < 0.05, ^##^*P* < 0.01 versus DEX group; ^&^*P* < 0.05, ^&⁣&^*P* < 0.01 versus BAY group; ^$^*P* < 0.05, ^$$^*P* < 0.01 versus sgNLRP3 group. One-way ANOVA followed by Tukey’s *post hoc* test **(B)**.

We measured protein levels of GR, NLRP3 inflammasome components, caspase-1 p20, IL-1β and IL-18 in HAPI cells (Figures 8A,B). Compared with the CON group, the expression of GR was significantly decreased in the DEX, BAY and sgNLRP3 groups. NLRP3 inflammasome components, caspase-1 p20, pro-IL-1β, IL-1β and IL-18 were significantly upregulated in the DEX group compared with the CON group. NLRP3 inflammasome components, caspase-1 p20, pro-IL-1β, IL-1β and IL-18 were significantly downregulated in the BAY group compared with the DEX group, while there was no significant difference from the CON group, except for NLRP3. ASC, pro-caspase 1, caspase-1 p20, pro-IL-1β, IL-1β and IL-18 were significant downregulated in the sgNLRP3 group compared with the DEX group, while there was no significant difference from the CON group, except for ASC and pro-IL-1β. Taken together, these results demonstrate that dexamethasone induces NF-κB p65 expression and nuclear translocation, which in turn activates the NLRP3 inflammasome and triggers an inflammatory cascade.

## Discussion

Numerous studies show that rats exposed to chronic restraint stress, consisting of a daily 6-h restraint period for 21 consecutive days, exhibit long-lasting depressive-like behavior (Donohue et al., 2006; Qiao et al., 2016). In the current study, we used this chronic restraint stress model of depression. Dexamethasone treatment of HAPI cells (a rat microglial cell line) was used to mimic depression and validate the *in vivo* findings. Firstly, we confirmed that chronic stress caused depression-like behavior in rats. Secondly, chronic stress induced excessive activation of the HPA (i.e., increased serum corticosterone concentration). Thirdly, increased glucocorticoid concentration may be the initial trigger of hippocampal microglia activation and neuroinflammation. Fourthly, chronic stress elevated ROS levels, upregulated NF-κB, and activated the NLPR3 inflammasome in the hippocampus. Finally, NF-κB inhibitors and NLRP3 knockout showed that NF-κB is a major activator of the NLRP3 inflammasome in microglia and the inflammatory cascade, which induce microglia neuroinflammation. Thus, the GR-NF-κB-NLRP3 signaling pathway in microglia triggers a cascade of downstream inflammatory factors that mediates chronic stress-induced hippocampal neuroinflammation and depressive behavior.

Chronic stress induces depression-like behaviors in animals, including anhedonia (reduced sucrose preference) (Chiba et al., 2012), which is a core symptom of human depression (Hill et al., 2012). Here, we found that exposure to chronic stress induced depressive-like behavior in the SPT, OFT and FST, which mainly showed reduced sucrose consumption (SPT), reduced total distance traveled, number of crossings and rearing (OFT), and increased immobility time (FST). These results are consistent with previous reports on the behavioral effects of chronic stress (Crider et al., 2018). Notably, we observed pathomorphological changes in the hippocampal regions. Therefore, chronic stress causes hippocampal damage that may underlie the depressive-like behavior.

Stress-induced glucocorticoid release is a neuroendocrine response to danger (Frank et al., 2013). The GR plays a major role in the adaptive stress response. However, stress-induced glucocorticoid hyper-secretion is associated with altered GR signaling (Finsterwald and Alberini, 2014). In the present study, we found that chronic stress exposure causes elevate corticosterone levels and a reduction in GR expression in the hippocampus. Studies have shown that SH-SY5Y cells exposed to dexamethasone mimic the hypersecretion of glucocorticoids in depression *in vitro* (Lim et al., 2018). Therefore, HAPI cells exposed to dexamethasone were used in this study to mimic glucocorticoid-induced neuroinflammation and depression. Dexamethasone treatment downregulated the GR. Both the *in vitro* and *in vivo* results suggest a link between chronic stress, glucocorticoids, GR signaling, and depression.

Overexposure to glucocorticoids may induce oxidative and inflammatory imbalance in the brain, thereby impairing neurogenesis and neuronal plasticity (Leonard and Maes, 2012). Corticosterone increases ROS levels in the brain, downregulates antioxidant enzymes, and induces depressive-like behavior (Sato et al., 2010). Our results that chronic stress increases levels of ROS in the hippocampus are in agreement with previous studies. Studies have also shown that chronic stress enhances NF-κB signaling induced by cholinergic depletion in the hippocampus, thereby increasing the expression of pro-inflammatory factors (Lee et al., 2018). This is in line with our current finding that chronic stress induces NF-κB expression and promotes NF-κB translocation to the nucleus in the hippocampus. Furthermore, dexamethasone increased levels of ROS and promoted NF-κB translocation to the nucleus in HAPI cells *in vitro*. Collectively, these results suggest that glucocorticoids signal through the GR to increase ROS levels and NF-κB nuclear translocation, promote transcription of downstream genes, and initiate the inflammatory response.

Microglia, the resident immune cells of the CNS, are markedly activated after stress exposed in brain regions associated with depression (Franklin et al., 2018). It has been reported that excessive activation of microglia increases the expression of inflammatory markers in the hippocampus of depressive individuals (Zhang Y. et al., 2016). Microglial activation is observed in the hippocampus after exposure to restraint stress in rodents (Tynan et al., 2010). In the present study, we discovered that microglia in the hippocampus were activated by chronic restraint stress, as shown by increased Iba-1 expression. Dexamethasone also similarly activated HAPI cells *in vitro*. Our findings are consistent with previous studies showing that chronic stress-induced depression is associated with increased numbers of Iba-1-positive cells in the hippocampus (Koo et al., 2018) and that activated microglia play an important role in neuroinflammation (Wang et al., 2018).

Accumulating evidence indicates that the NLRP3 inflammasome is involved in neuroinflammation, neurodegenerative disorders (Jang et al., 2016; Zhang P. et al., 2016) and neuropsychiatric disorders such as depression (Jeon et al., 2017). Studies show that both NF-κB and ROS can activate the NLRP3 inflammasome. NF-κB translocates to the nucleus binding with DNA and triggers the transcription of pro-IL-1β, pro-IL-18 and NLRP3 (Choi and Ryter, 2014). ROS triggers the assembly of the NLRP3 inflammasome complex by recruiting ASC and pro-caspase 1 (Hu and Chai, 2016), driving a pro-inflammatory response that ultimately leads to cell damage (Gurung et al., 2015). *In vivo* results indicate that chronic stress activates NLRP3 inflammasome, which are expressed as NLRP3 expression, caspase-1 cleavage, and subsequent IL-1β and IL-18 generation. *In vitro* studies also show that dexamethasone activates NLRP3 inflammasome in HAPI cells. Interestingly, a recent study showed NF-κB was reported to be an ROS-sensitive transcription factor that mediated NLRP3 inflammasome activation (Li et al., 2014). Another study showed that NLRP3 deficiency significantly abolishes depressive behavior in mice under immobilization stress (Alcocer-Gomez and Cordero, 2014).

To further clarify the roles of NF-κB and the NLRP3 inflammasome in depression, we inhibited NF-κB and knocked out the NLRP3 gene *in vitro*. Western blot results confirmed successful knockdown of NLRP3 gene (Supplementary Figure S1). Downregulation of NF-κB and NLRP3 reduced ROS levels, inhibited microglial activation, decreased Iba-1 expression, inhibited NF-κB nuclear translocation, and reduced NLPR3 inflammasome activation. These findings indicate that ROS and NF-κB-induced activation of the NLRP3 inflammasome in microglia contributes to hippocampal injury and the development of depression. Consistent with previous study, activated NLRP3 inflammasomes in microglia are involved in CMS-induced depression-like behavior in rats (Wang et al., 2018). Therefore, microglia and the NLRP3 inflammasome may have potential as drug targets for the treatment of depression. A better understanding of the anti-inflammatory properties of antidepressants may help advance therapy for neurodegenerative and psychiatric diseases associated with microglial hyperactivation.

In summary, HAPI cells were pretreated with dexamethasone to assess stress-induced changes in microglia cells in culture. Compared with *in vivo* results, it was first demonstrated that increased glucocorticoid concentration is the initial trigger for microglial activation and neuroinflammation. Secondly, it was proved that NF-κB is function as the upstream transcriptional activator of NLRP3. Finally, it was further confirmed that NLRP3 inflammasome activation in microglia mediates chronic stress-induced hippocampal neuroinflammation.

There are a number of limitations to this study. First, only limited animal models can properly express psychiatric symptoms (Suzuki et al., 2019), although chronic restraint stress has been widely used to induce depression and anxiety-like behavior in animal models (Huang et al., 2015), it may not adequately mimic depression in humans. Second, the production and release of IL-1β and IL-18 are regulated by active caspase-1. However, the activation of caspase-1 is promoted by various NLR family members, including NLRP1, NLRP3, NLRC4 (Eder, 2009). Further study is therefore needed to evaluate the impact of other inflammasome subtypes on the activation and release of IL-1β and IL-18. In our study, we only focus on the role of NLRP3 in the process of depression.

## Conclusion

**FIGURE 9:**
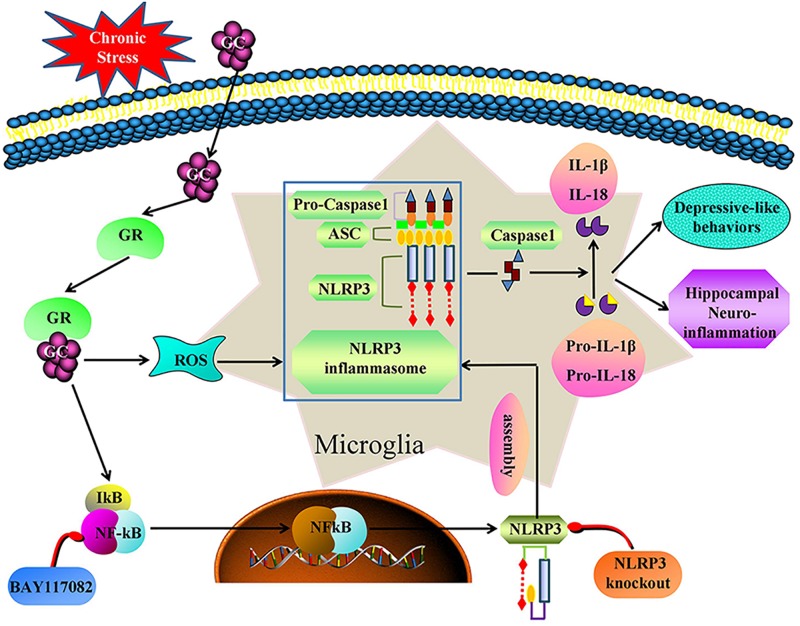
Illustration for mechanism of chronic stress induces hippocampal neuroinflammation and depression-like behavior. Chronic stress activation the GR-NF-κB-NLRP3 signal pathway in hippocampal microglia and eventually mediates cascade reaction of inflammatory factors (IL-1β and IL-18). Glucocorticoids was an initial activator of NF-κB-NLRP3 signal pathway. The main effective inhibitors of this signal pathway are marked in red. GC, glucocorticoid; GR, glucocorticoid receptors; ROS, Reactive oxygen species; NF-κB, nuclear factor-κB; NLRP3, NOD-like receptor protein 3; ASC, Apoptosis-associated speck-like protein containing a CARD; IL-1β, Interleukin-1β; IL-18, Interleukin-18.

We demonstrate that, chronic stress causes hippocampal neuroinflammation and depression-like behavior by activating the GR-NF-κB-NLRP3 signaling pathway in microglia (summarized in Figure 9). Our study provides fresh insight into the pathogenesis of depression and provides new therapeutic targets for the treatment of the disease. Inhibiting glucocorticoid release, NF-κB nuclear transcription, NLRP3 inflammasome activation and restoring microglial homeostasis in the hippocampus are novel strategies for the development of effective treatments for the management of depression in humans.

## Data Availability

All datasets used to support the results of this study are included in the manuscript without any reservations.

## Ethics Statement

Animal Subjects: The animal study was reviewed and approved by the ethical committee of Northeast Agricultural University (SRM-11), and experiments were carried out in accordance with the National Institutes of Health Guide for Care and Use of Laboratory Animals.

## Author Contributions

HF and XF contributed to study design. XF, YZ, and CW carried out the experiment. MS, YY, and TY contributed to data statistical analysis. XF wrote the manuscript. All authors read and approved the final manuscript.

## Conflict of Interest Statement

The authors declare that the research was conducted in the absence of any commercial or financial relationships that could be construed as a potential conflict of interest.
