# Supplementary material for: Glucocorticoid-Driven NLRP3 Inflammasome Activation in Hippocampal Microglia Mediates Chronic Stress-Induced Depressive-Like Behaviors
Source: Front Mol Neurosci. 2019 Aug 29;12:210. doi: 10.3389/fnmol.2019.00210 (PMC6727781; doi:10.3389/fnmol.2019.00210)
Supplement: Supplementary file 1 [file Table_1.DOCX]

**Supplementary Figure**


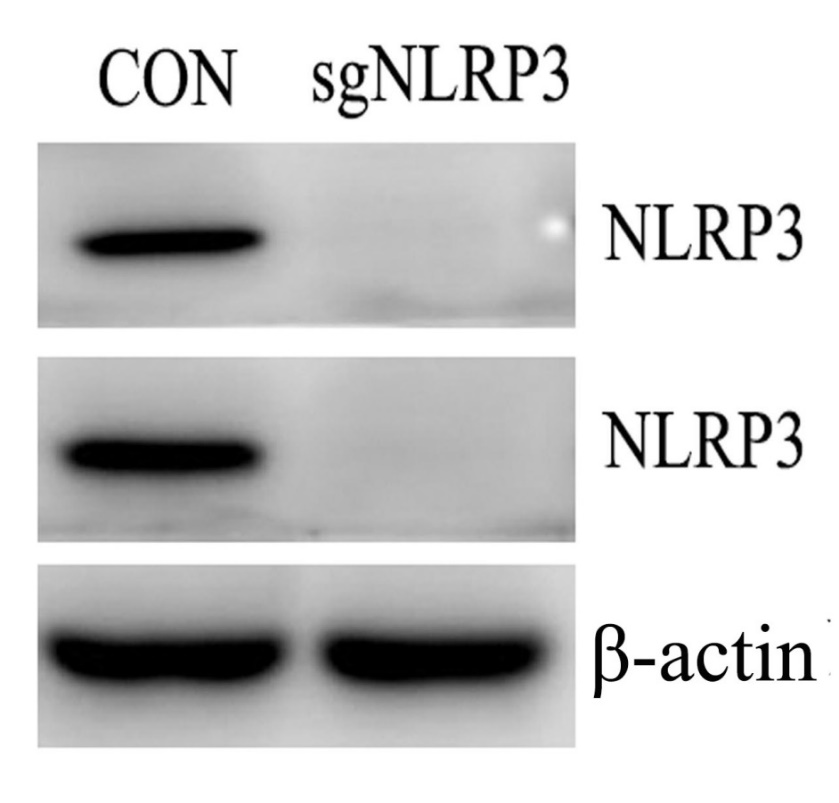


**Supplementary Figure 1.** NLRP3 was successfully knocked out by CRISPR/Cas9 techniques. NLRP3 knockdown was created by CRISPR/Cas9 and evaluated by Western blot. β-actin was used as a loading control
